# The Chemistry of Curcumin: From Extraction to Therapeutic Agent

**DOI:** 10.3390/molecules191220091

**Published:** 2014-12-01

**Authors:** Kavirayani Indira Priyadarsini

**Affiliations:** Radiation & Photochemistry Division, Bhabha Atomic Research Centre, Mumbai 400085, India; E-Mail: kindira@barc.gov.in; Tel.: +91-22-25595399

**Keywords:** curcumin, extraction, synthesis, degradation, metal chelation, nanoformulation

## Abstract

Curcumin, a pigment from turmeric, is one of the very few promising natural products that has been extensively investigated by researchers from both the biological and chemical point of view. While there are several reviews on the biological and pharmacological effects of curcumin, chemistry reviews are comparatively scarcer. In this article, an overview of different aspects of the unique chemistry research on curcumin will be discussed. These include methods for the extraction from turmeric, laboratory synthesis methods, chemical and photochemical degradation and the chemistry behind its metabolism. Additionally other chemical reactions that have biological relevance like nucleophilic addition reactions, and metal chelation will be discussed. Recent advances in the preparation of new curcumin nanoconjugates with metal and metal oxide nanoparticles will also be mentioned. Directions for future investigations to be undertaken in the chemistry of curcumin have also been suggested.

## 1. Introduction

Scientific research spanning over more than four decades has confirmed the diverse pharmacological effects of curcumin and established its ability to act as a chemopreventive agent as well as a potential therapeutic agent against several chronic diseases [1,2,3,4,5,6]. Curcumin, having nearly a two centuries old scientific history, is still attracting researchers from all over the world. Starting from 1815, when curcumin was first isolated from turmeric, there were only a few reports till the 1970s on its chemical structure, synthesis, biochemical and antioxidant activity [7,8]. However after the report by Aggarwal and co-workers [9] in 1990s on its potential anticancer effect, the pace of curcumin research has grown rapidly, with more than 14,000 citations on curcumin to date. While the majority of researchers have been pursuing the biological aspects, a few others were interested in understanding the important chemistry of curcumin behind its unique biological activity. Curcumin research has become one of the most favorite subjects for all the branches of chemistry, including organic, inorganic, physical and analytical chemists. In organic chemistry the extraction and synthesis of curcumin and new synthetic derivatives was the main focus of research. Inorganic chemists have used its metal chelating abilities through the β-diketo group to form new structural entities with modified biochemical activities. Physical chemists have focused on the highly sensitive spectroscopic properties of curcumin to study its interactions with microheterogeneous systems and biomolecules. Analytical chemists have been employing curcumin’s unique absorption spectroscopic properties to identify and quantitatively estimate trace elements like for e.g., estimation of boron, as a red colored product [10]. Other chemistry studies that are useful in understanding the biological activity of curcumin are its chemical reactivity with reactive oxygen species (ROS), addition reactions, degradation and formation of nanoconjugates and formulations. Considering the number of reviews in many related fields of curcumin, in the present manuscript, only some of the important recent findings in curcumin chemistry have been reviewed that have contributed to its potential applications in the development of curcumin- based drugs.

## 2. Extraction of Curcumin from Turmeric and Detection

*Curcuma longa* L (turmeric) is cultivated in tropical and subtropical regions. The largest worldwide producer of turmeric is India, where it has been used as a home-remedy for several ailments for ages [1,2,3,4]. Depending on its origin and the soil conditions where it is grown, turmeric contains 2%–9% curcuminoids. The word “curcuminoid” indicates a group of compounds such as curcumin, demethoxycurcumin and bis-demethoxycurcumin and cyclic curcumin. Out of these, curcumin is the major component, and cyclic curcumin is the minor component.

Although extraction and separation of curcumin from turmeric powder was reported way back in 1815, more improved and advanced extraction methods are still being reported, even after two centuries [11,12,13,14,15,16,17,18,19,20,21,22,23,24,25,26,27]. Solvent extraction followed by column chromatography has been the most commonly employed method reported for separating curcumin from turmeric, and several polar and non-polar organic solvents have been used, including hexane, ethylacetate, acetone, methanol, *etc.* Of the organic solvents employed, ethanol has been found to be the most preferred solvent for extracting curcumin. Although chlorinated solvents extract curcumin very efficiently from turmeric, they are not commonly employed due to their non-acceptability in the food industry. Soxhlet extraction, ultrasonic extraction, microwave, zone-refining and dipping methods have been tried, and among these the Soxhlet, ultrasonic and microwave extractions are the most commonly employed methods [11,12,13,14,15,16]. Recently pulse ultrasonic and microwave-assisted extraction methods have also been reported to be better than the continuous methods [14]. Increasing the temperature in the range of 60 to 80 °C has been found to improve the extraction [16]. With its increasing use in dietary supplements, researchers are developing extraction methods employing food grade solvents like triacylglycerols to give good yields [17]. Another commercially viable and efficient extraction method is using supercritical carbon dioxide [18,19]. Being free from organic solvents, pilot plants based on supercritical carbon dioxide have been established in several countries for the extraction of curcumin from turmeric. Normal operating conditions for this are at pressures between 25 to 30 MPa and a temperature of 318 K. There are also a few reports on enzyme-assisted extraction, where pretreatment of turmeric with enzymes like α-amylase and glucoamylase yielded significant increases in curcumin yield [20]. However due to increase in the cost of extraction, this method is not commercially viable.

Curcumin can be separated from curcumin mix (a mixture of curcumin, demethoxycurcumin and bisdemethoxycurcumin) by column chromatography by adsorbing the mixture on silica gel using mixtures of solvents like dichloromethane/acetic acid or methanol/chloroform to yield three different fractions. The curcumin fraction is further purified on silica gel using chloroform/dichloromethane and ethanol/methanol mixtures as eluents [3,4,21,22,23,24]. Methods for the detection and estimation of curcumin have mostly employed the high performance liquid chromatography (HPLC) technique. In general reverse phase C18 columns are used as stationary phase and different gradients of solvents containing acetonitrile/water or chloroform/methanol have been employed as the mobile phase [21,22,23,24]. For detection of curcumin, absorption detectors in the wavelength range from 350 to 450 nm range or in the UV region using a common detection wavelength in the range of 250 to 270 nm is simple and extremely useful. HPLC-diode array and fluorescence detection methods have also been used by several researchers. Liquid chromatography-coupled mass spectrometry has been another versatile tool for detecting curcumin. Of all these the most sensitive methods for detection of curcumin (up to 1 ng/mL) is by fluorescence, by exciting in the 400 to 450 nm region. High-performance-thin layer chromatography methods using aluminium plates precoated with silica gel as stationary phase and chloroform-methanol as solvent have been very useful for both detection and separation. Ali *et al.* [22] described efficient separation and detection of curcumin in turmeric powders employing a HPLC method using a phenyl column and acetonitrile-methanol-water as the mobile phase. Microemulsion electrokinetic chromatography using oil droplets and surfactants, has been found to be good for both extraction and estimation of curcumin in food and medicinal samples [25]. Capillary electrophoresis with amperometric detection can be routinely employed to estimate curcumin/turmeric in food materials [26]. HPLC and LC/MS have been found to detect low quantities of curcumin in biofluids to evaluate the pharmacokinetics, biodistribution and metabolism [16]. Ultraperformance liquid chromatography (UPLC) coupled with online tandem mass spectrometry has been used to detect curcumin metabolites in plasma and urine, with detection limits of 2.5 ng/mL [27].

## 3. Synthesis of Curcumin

A century after its isolation from turmeric, the first paper on synthesis of curcumin was reported by Lampe in 1918 [28]. The method involved five steps starting from carbomethoxyferuloyl chloride and ethyl acetoacetate. Later Pabon [29] reported a simple method for the synthesis of curcumin in high yields using acetyl acetone and substituted aromatic aldehydes in the presence of boron trioxide (B_2_O_3_), trialkyl borate and *n*-butylamine and with slight modifications this method by Pabon [29] has been adopted by several research groups for practically all subsequent curcumin syntheses [3,4,30,31,32]. There are some patents indicating utilization of B_2_O_3_, trialkylborate and *n*-butylamine along with inert organic amide solvents to improve the yields. Attempts to replace boric oxide with boric acid did not prove to be successful. Rao and Sudheer [32] proposed the use of trifluoroboronite and produced stable curcuminoid trifluorboronites that can be hydrolysed in aqueous methanol at pH 5.8 to get curcumin.

**Scheme 1:**
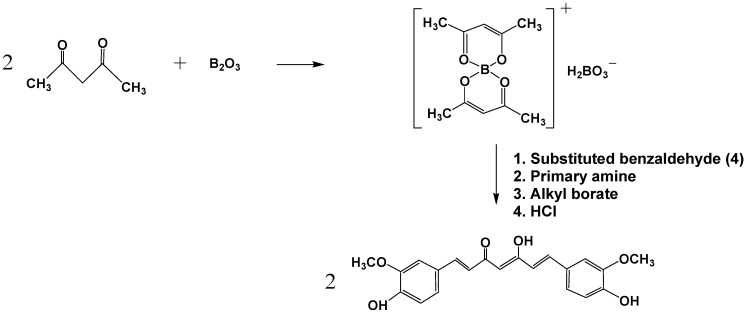
Synthesis of curcumin by the general method proposed by Pabon [29].

In all these methods the primary step is the reaction of 2,4-diketones with suitably substituted aromatic aldehydes. To prevent participation of the diketone in Knoevenagel condensations, it is complexed with boron. Anhydrous conditions and polar aprotic solvents, where curcumin can be separated easily from the reaction mixtures, are suitable for these reactions. Primary and secondary amines are used as catalysts to provide the necessary basicity to deprotonate the alkyl groups of the diketone. To remove the water produced during the condensation reaction scavengers like alkyl borates are employed. Unless removed, the water can react with the diketone complex, thereby reducing the curcumin yield. The boron complex dissociates into curcumin under slightly acidic conditions. Curcumin from this reaction mixture can be separated by washing and repeated precipitation followed by column chromatography. The overall reaction scheme following the method by Pabon is given in Scheme 1.

## 4. Structural Characteristics of Curcumin

**Scheme 2:**
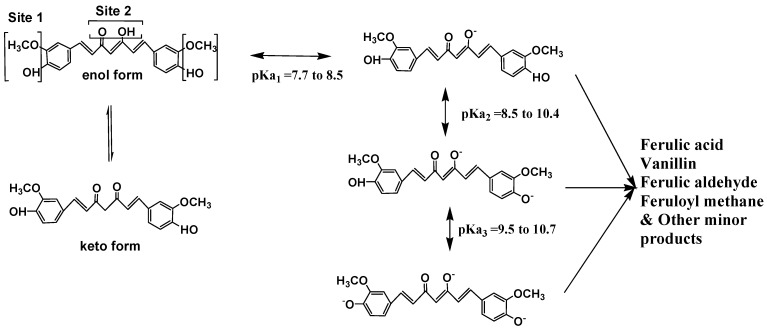
Keto-enol tautomerism, Prototropic equilibria and degradation products of curcumin.

Curcumin is a symmetric molecule, also known as diferuloyl methane. The IUPAC name of curcumin is (1*E*,6*E*)-1,7-bis(4-hydroxy-3-methoxyphenyl)-1,6-heptadiene-3,5-dione, with chemical formula C_21_H_20_O_6_, and molecular weight of 368.38. It has three chemical entities in its structure: two aromatic ring systems containing o-methoxy phenolic groups, connected by a seven carbon linker consisting of an α,β-unsaturated β-diketone moiety [3,4,5,6,33]. The chemical structure of curcumin is given in Scheme 2.

The diketo group exhibits keto-enol tautomerism, which can exist in different types of conformers depending on the environment [33]. In the crystal state it exists in a *cis*-enol configuration, where it is stabilized by resonance assisted hydrogen bonding and the structure consists of three substituted planar groups interconnected through two double bonds. In most of the non-polar and moderately polar solvents the enol form is generally more stabilized than the keto form by 5 to 8 kcals/mol depending on the nature of the solvent. Due to extended conjugation, the π electron cloud is all along the molecule. In solution it exists as *cis-trans* isomers where the *trans*-form in which the two phenolic-methoxy groups are on the opposite sides of the curcumin backbone is slightly more stabilized than the *cis-*form, where the phenolic methoxy groups are in the same side up the backbone. The computed dipole moment of curcumin in the ground state is 10.77 D. It is a hydrophobic molecule with a logP value of ~3.0. It is almost insoluble in water and readily soluble in polar solvents like DMSO, methanol, ethanol, acetonitrile, chloroform, ethyl acetate, *etc.* It is sparingly soluble in hydrocarbon solvents like cyclohexane and hexane. The absorption spectrum of curcumin has two strong absorption bands, one in the visible region with maximum ranging from 410 to 430 nm and another band in the UV region with maximum at 265 nm region. The molar extinction coefficient of curcumin in methanol is 55,000 dm^3^·mol^−1^·cm^−1^ at 425 nm. Curcumin is a weak Brönsted acid, with three labile protons, and accordingly three pK_a_s have been estimated corresponding to three prototropic equilibria (Scheme 2). Both NMR and absorption spectrometry have been used to estimate the pK_a_. The first pK_a_ in the pH range of 7.5 to 8.5 changes curcumin from yellow to red. The chemical reactivity and solubility of the anionic curcumin, *i.e.*, in the basic pH range increases and this form of curcumin is more water soluble than the neutral form. The absorption maximum of fully deprotonated (red in colour) curcumin in alkaline pH (>pH 10) is at 467 nm and the molar extinction coefficient is 53,000 dm^3^·mol^−1^·cm^−1^. There is still a debate which one of the three, *i.e.*, the enolic OH or the phenolic OH is the most acidic. Although calculations indicate that the enolic OH is the most acidic group, the pH dependent spectral changes are difficult to distinguish between the two protons. From ^1^H-NMR studies Borsari *et al.* [34] proposed the pK_a_ of 12.5 for the deprotonation of the enolic proton and another pK_a_ at 13.6 for the phenolic protons. These values for enolic protons however differ significantly from those reported by other methods. With the availability of other spectroscopic techniques, it should be possible to resolve these differences in the estimation of pK_a_s in the future.

Aqueous curcumin solutions can be prepared by adding surfactants, lipids, albumins, cyclodextrins, biopolymers *etc.* Micellar solutions using surfactants are the best suited for preparing high concentration curcumin solutions in water [33]. However while using aqueous surfactant solutions in biological systems, care must be taken by performing proper control experiments, as surfactants can interfere in biological studies.

## 5. Curcumin Reactivity

Curcumin has three reactive functional groups: one diketone moiety, and two phenolic groups [6]. Important chemical reactions associated with the biological activity of curcumin are the hydrogen donation reactions leading to oxidation of curcumin, reversible and irreversible nucleophilic addition (Michael reaction) reactions, hydrolysis, degradation and enzymatic reactions. All these have significant role in different biological activities of curcumin. They are discussed briefly.

### 5.1. Reactions with ROS

**Scheme 3:**
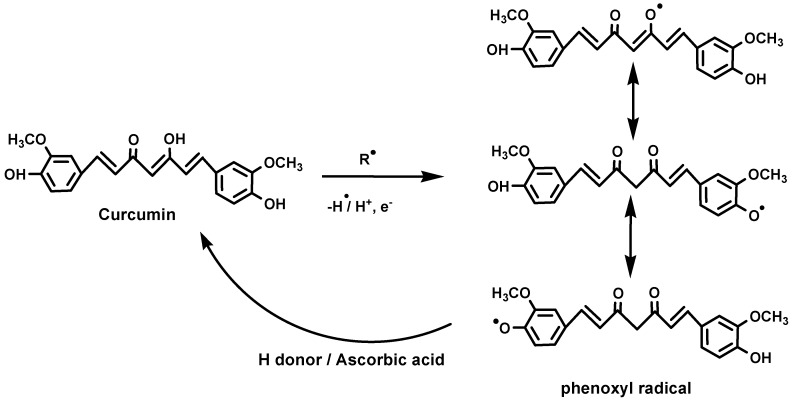
Possible sites of attack of free radical oxidants with curcumin and stabilization of phenoxyl intermediate and its regeneration by ascorbic acid.

Curcumin has been found to be an excellent scavenger of most ROS, a property that bestows curcumin with antioxidant activity in normal cells. ROS consists of both free radical oxidants and molecular oxidants [33,35,36,37,38,39,40,41]. Free radical oxidants participate in hydrogen abstraction and also in electron transfer reactions. All three active sites of curcumin can undergo oxidation by electron transfer and hydrogen abstraction. Detailed investigations by different groups have confirmed that during free radical reactions, the most easily abstractable hydrogen from curcumin is from the phenol-OH group, resulting in formation of phenoxyl radicals, which are resonance stabilized across the keto-enol structure (Scheme 3).

For example the reaction of peroxyl radicals with curcumin produces curcumin phenoxyl radicals, which are less reactive than the peroxyl radicals and thereby cause protection from ROS-induced oxidative stress [34,35,38]. The regeneration reaction of phenoxyl radicals back to curcumin by water soluble antioxidants like ascorbic acid, impart the molecule with a chain breaking antioxidant ability like vitamin E [38]. Reported in the literature are scavenging reactions of several other free radical ROS such as hydroxyl radicals, superoxide radicals and alkoxy radicals by curcumin [36,37,38,39]. The reaction of curcumin with superoxide radicals has been found to be as efficient as well known lipid soluble antioxidants and the reaction also leads to catalytic degradation of superoxide in which curcumin acts as a superoxide dismutase mimic [37].

Among the molecular oxidants, reactions with peroxynitrite, hydrogen peroxide are the most common ones. In several biological models curcumin has been found to protect cells under conditions where there is excessive production of these molecular oxidants. However there are not many studies elucidating the possible chemical reactions and identification of the reaction products. There are few reports in the literature on direct reaction of curcumin with peroxynitrite [40,41]. The reported rate constants and the inhibition concentrations of curcumin to prevent nitrotyrosine formation indicate that curcumin is as powerful antioxidant against peroxynitrite-induced oxidative stress.

### 5.2. Chemical Degradation and Metabolism

Curcumin undergoes chemical degradation in aqueous-organic solutions and the degradation increases as the pH is increased, which is of a serious concern in its applications [6,33,42,43,44,45]. Most phenols in solution form polymers over time, but the degradation of curcumin is not through the phenolic group but is rather found to be through the α,β-unsaturated β-diketo moiety. In dilute solutions (*i.e*., in micromolar solutions) 90% curcumin degrades in 30 minutes. However the percentage degradation will decrease at high concentrations. Several important products have been identified as a result of curcumin degradation. They are *trans*-6(4'-hydroxy-3'-methoxyphenyl)-2,4-dioxo-5-hexanal, ferulic aldehyde, ferulic acid, feruloylmethane and vanillin (Scheme 2). Although not fully understood, it is believed that the degradation is by hydrolysis through the diketo moiety. However the degradation is significantly decreased when curcumin is attached to lipids, liposomes, albumins, cyclodextrin, cucurbituryl, surfactants, polymers and many other macromolecular and microheterogenous systems [33]. Thus has been found to be of great use that stable curcumin solutions could be prepared in culture medium containing 10% Fetal Bovine Serum (FBS) and also in human blood.

Curcumin undergoes much faster degradation when exposed to sunlight [33,46]. It is one common observation that curcumin/turmeric stains can be quickly removed on exposure to sunlight The colorless products identified during photodegradation of curcumin are vanillin, ferullic acid, and other small phenols, indicating a similar product distribution during photochemical degradation as in chemical degradation in solution. This photodegradation involves formation of the excited states of curcumin. Some reports indicate that curcumin generates singlet oxygen and other ROS on photoexcitation and this is actually responsible for the photobiological and photodynamic activity of curcumin [47]. In such case the degradation of curcumin after photoexcitation must proceed though the triplet excited state of curcumin [33]. Photophysical studies reported the lifetime of triplet excited state of curcumin to be in microseconds, suggesting that the degradation may proceed very fast and compete with singlet oxygen formation. The photodegradation is accelerated in presence of TiO_2_ nanoparticles, and this method can be employed to remove turmeric stains from cotton fabrics [48].

The metabolism of curcumin in rats and humans produces different products [49,50,51,52,53]. Two major pathways have been identified in curcumin metabolism, like O-conjugation and reduction. The O-conjugation products are curcumin glucuronide and curcumin sulfate. The reduction products are tetrahydrocurcumin, hexahydrocurcumin and octahydrocurcumin. Other minor products are dihydrocurcumin glucuronide, tetrahydrocurcumin glucuronide, ferulic acid and dihydroferulic acid. The formation of these products has been confirmed by HPLC and mass spectrometry. Although it has been reported that these processes occur enzymatically, the exact enzymes involved in all these specific reaction products are still a matter of debate. Sulfonation of curcumin through human phenol sulfur transferase enzymes and the formation of reduction products through alcohol dehydrogenase is proposed. Comparing these metabolic products with the degradation products, it appears that simple hydrolytic degradation is prevented in biofluids. Since the degradation may occur through the β-diketo structure, one can presume that in these systems curcumin is not in free form but rather in conjugated form bound to some proteins or other biomolecules, and as the diketo moiety is involved in binding to the proteins [5,6], it is not available for hydrolytic degradation. It may also be implied that the specific enzymatic reactions are probably much faster and do not allow the slow hydrolytic degradation, therefore the latter process cannot compete with the former reaction. This leaves a bigger challenge for chemists to understand the differences between degradation and metabolic reactions in terms of kinetic parameters and also identify the crucial mechanism in these reactions.

### 5.3. Nucleophillic Addition Reactions of Curcumin

The α,β-unsaturated β-diketo moiety of curcumin participates in nucleophilic addition reactions. This reaction, known as the Michael addition, occurs between the unsaturated ketone as an acceptor and anions of –OH, –SH, –SeH as donors. It is a 1,4-addition reaction and the resultant product formations are mostly irreversible, but they can be made reversible under oxidizing and basic conditions. Since the anions only act as nucleophiles, pH conditions are very important for this reaction to take place. At physiological pH both –OH and –SH are protonated but –SeH can easily undergo deprotonation, therefore it acts as a better nucleophile. This reaction has been reported to be extremely useful to explain the biological chemistry of curcumin in living cells [3,5,6,54,55,56,57]. Of special interest has been the reaction of biological thiols like glutathione having –SH groups [54,55]. Indeed curcumin-glutathione conjugates have been isolated in different systems. Formation of this addition product would lead to the depletion of the intracellular glutathione levels in cells, thereby leading to reduction in the overall antioxidant defense. Although a few reports suggest that this is a reversible reaction, it is not yet confirmed, under the conditions present in living cells, whether such reaction is reversible or not. Reversibility of the reaction can be expected under oxidizing conditions and at basic pH.

**Scheme 4:**
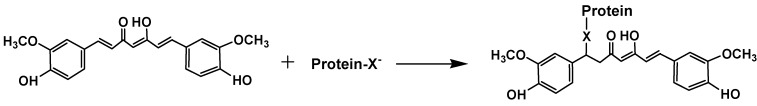
Michael addition products of curcumin with protein thiols and selenols where, X = S or Se.

A similar reaction has been observed during the inhibition of thioredoxin reductase by curcumin [5,6,56]. Thioredoxin reductase is a crucial enzyme involved in maintaining cellular redox homeostasis. The active centre in this enzyme is selenocysteine. The selenol of selenocysteine, being a stronger nucleophile at physiological pH, easily undergoes 1,4-addition with curcumin, forming covalently bonded species. This reaction is speculated to be mainly responsible for the effective inhibition of the thioredoxin reductase enzyme by curcumin. Scheme 4 shows the structure and the chemical reaction product of curcumin with protein thiols and selenols by Michael addition.

The methylenic hydrogen of the diketo/enol moiety of curcumin can also act as a nucleophile and participate in Michael addition reactions with stronger electrophiles [58], but such reactions may not have significance in biological systems.

Chemically modified curcumin derivatives have been prepared by condensation/addition reactions like e.g., semicarbazone derivatives and oxime derivatives of curcumin [59,60]. These stable products prepared independently have been examined for anti-cancer activity. In most of these studies, it has been reported that these derivatives are more cytotoxic to cancer cells than free curcumin. This prompts us to speculate that probably the glutathione conjugate of curcumin may also act as a cytotoxic agent and contribute to the overall anti-tumor activity of curcumin.

## 6. Chemistry of Curcumin-Metal Ion Interactions

**Figure 1:**
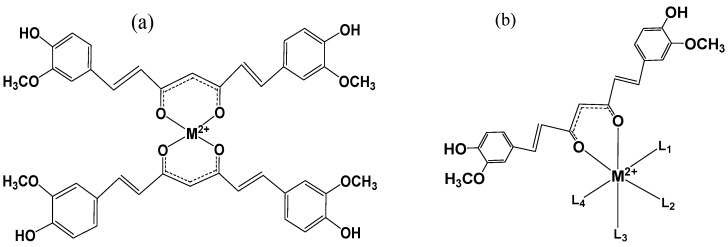
(**a**) Structure of 2:1 curcumin:metal complex; (**b**) Mixed ligand curcumin:metal complex.

Curcumin forms strong complexes with most of the known metal ions. The α,β-unsaturated β-diketo moiety of curcumin is an excellent chelating agent. In the last one and half decades, many papers have been published on metal-curcumin complexes [61,62,63,64,65,66,67,68,69,70,71,72,73,74,75,76,77,78,79,80,81,82,83,84,85,86,87,88,89,90,91,92,93,94,95]. Curcumin is a monobasic bidentate ligand and forms stable complexes with almost all the metals and non-metals. In general, stable structures with 2:1 (ligand:metal) stoichiometry are observed. There are very few reports on 3:1 ligand:metal complexes, e.g., an octahedral complex reported with Fe^3+^ [64]. Metal coordination of curcumin occurs through the enolic group, where the enolic proton is replaced by the metal ion and the *o*-methoxy phenolic moiety remains intact in the complexes. The metal-oxygen bond is characterized by IR spectroscopy signals at 455 cm^−1^ and the carbonyl peaks in the complexes show a small shift of ~10 cm^−1^ on coordination to metals. Changes in NMR chemical shifts of curcumin have also been reported on metal coordination. The shifts however depend on the affinity and thermodynamic stability of the resulting complexes. In the case of strong complexes, the resonances of protons attached to the double bonds of the alkyl chain show significant downfield shifts, while the enolic protons show negligible shifts in the ^1^H-NMR spectra [34], and the ^13^C-NMR spectrum shows down- and up-field shfts of carbons near the coordination site. There are several papers published in the literature on complexes of curcumin with transition metals like Fe^3+^, Mn^2+^, Ni^2+^, Cu^2+^, Zn^2+^, Pb^2+^, Cd^2+^, Ru^3+^, Re^3+^ and many others. Complexes with non-transition metal ions and rare earth ions like Al^3+^, Ga^3+^, Sm^3+^, Eu^3+^, Dy^3+^, Y^3+^, Se^2+^ and metal oxides like VO^2+^ have also been synthesized. The structure and physical properties of these complexes depend on the nature of the metal ion, as well as the stoichiometry of the reaction conditions, which in turn decides their stability and reactivity. Stable 2:1 complexes of some transition metals can be prepared by mixing stoichiometric amounts of curcumin and metal salts in suitable organic solvents and refluxing for few hours, the complex can be separated as a precipitate, and purified either by column chromatography and repeated crystallization. The general chemical structure of 2:1 complexes of curcumin with metals is given in Figure 1a.

Curcumin-metal complexes not only modify the physico-chemical properties of curcumin but they also affect the biological reactivity of the metals. In general it has been observed that complexation with curcumin reduces the toxicity of the metals and some curcumin complexes with metals like Cu^2+^, Mn^2+^, act as new metal-based antioxidants [67,68,69,70,71,72,73,74]. Due to the reversible electron transfer reactions with superoxide ions, Cu^2+^ and Mn^2+^ complexes of curcumin act as superoxide dismutase enzyme mimics. Metal complexes of curcumin have greater significance in view of the pathology of Alzheimer’s disease, where it has been found that due to its lipophilic nature, curcumin can cross the blood brain barrier and chelate metal ions that are toxic to the neurons. It has also been observed that the incidence of Alzheimer’s disease is significantly reduced among people that are known to regularly consume turmeric in their diet. Curcumin forms stable complexes with all the metals involved in Alzheimer’s disease [74,75,76]. The interaction of curcumin with Al^3+^, earlier considered to be responsible for development of Alzheimer’s disease, has been studied extensively. Curcumin forms three different types of complexes with Al^3+^, depending on the stoichiometry of the reaction. The 1:1 Al^3+^-curcumin complex showed less affinity to DNA binding than free Al^3+^, which has been attributed to its ability to reduce development of Al^3+^ induced Alzheimer’s disease [75,76]. Many other applications of curcumin metal complexes have been reported. Ga^3+^ curcumin complexes are being developed as innovative bioceramics [77]. Zn^2+^-curcumin complexes showed anti-cancer, gastroprotective and antidepressant effects in rats [78,79]. *In vivo* antiarthritic activity was reported for five co-ordinated curcumin-gold (Au^3+^) complexes [80]. Vanadyl-curcumin (VO(Cur)_2_)^2+^ complexes show antioxidant and anti-rheumatic activity [81]. Through metal co-ordination, curcumin reduces the toxicity of heavy metals like Hg^2+^, Cd^2+^, Pb^2+^ where significant reduction in heavy metal-induced oxidative stress is reported through complex formation [82,83,84,85,86].

Owing to their positive charges, most of the metal complexes of curcumin bind to DNA [66,78]. Some reports indicate that due to binding, the curcumin-metal complexes induce DNA damage and thereby exhibit pro-oxidant behavior. Accordingly, curcumin-metal complexes are also being explored as better anti-tumor agents than curcumin itself [87,88]. Therefore there is still some confusion about the role of metal-curcumin complexes, in biology whether they act as antioxidants or pro-oxidants. It appears that some metal complexes act as antioxidants, while some others may be pro-oxidants. This antioxidant/pro-oxidant activity of a metal complex depends on several factors such as nature of metal ion, co-ordination number, structure, stability and electrochemical potential of the complex. More systematic classification and chemistry of metal-curcumin complexes is necessary in the future. Biological evaluation of these complexes is more difficult in *in vivo* systems, as the bioavailabilty of most of the curcumin-metal complexes is very low and the experiments are difficult to perform due to the lower solubility either in organic solvents like DMSO or in biological fluids. However, it is beyond any doubt that this *in vivo* complexation of curcumin with metals plays a significant role in reducing metal-induced toxicity.

Recently a few mixed ligand complexes of curcumin with unique chemical and biological activities have been reported. In these complexes (Figure 1b), the curcumin to metal ratio is 1:1 [90,91,92,93]. A few examples of these mixed ligand complexes are summarized here. Porphyrin-bridged curcumin complexes of Cu^2+^, Ni^2+^ and Zn^2+^ showed improved photodynamic activity in plasmid DNA models [90]. Complexes of curcumin-and 4,4'-bipyridine with Zn^2+^ were more effective than curcumin to kill neuroblastoma cells. Curcumin-terpyridyl-lanthanum (La^3+^) complexes showed enhanced photocytotoxicity in HeLa cells [91]. Bipyridyl-curcumin complexes of Pd^2+^ inhibit the growth of human prostate cancer cells [87]. Mixed ligand-curucumin complexes with rare earth metals like Sm^3+^, Eu^3+^ and Dy^3+^ showed antibacterial activity [92,93].

The absorption spectrum of curcumin is altered on complexation with metals. The 1:2 transition metal complexes of curcumin showed a blue shift of the absorption maximum [33,70,71]. The fluorescence quantum yield of curcumin is drastically reduced on metal chelation. However the Al^3+^ complexes and rare earth metal complexes of curcumin are more fluorescent [76,92,93] and also in mixed ligand complexes, reports suggest that the fluorescence of curcumin remains unaffected. Fluorescent curcumin-metal complexes are being explored for imaging of cancer cells. Rare earth complexes of curcumin and pyridine exhibit two-photon absorption in the wavelength range 700 to 800 nm and such complexes have been used for the imaging of MCF-7 cells [93]. Re(CO)_3_(Curcumin)(H_2_O) complexes are fluorescent and show affinity to beta-amyloid plaques, which has potential to be explored in microscopic imaging of the tissue of Alzheimer’s disease patients [94]. Similarly ^99^T(CO)_3_(curcumin)(H_2_O) complexes have been produced in high (>90%) radiochemical yield, and showed significant affinity to β-amyloid plaques and such systems are being developed as novel radiodiagnostic agents for Alzheimer’s disease [94,95]. Recently 2:1 complexes of curcumin with ^68^Ga^3+^ have been prepared with high radiochemical purity. These compounds have also been reported to be binding to β-amyloid fibrils very strongly [95], with possible applications in Alzheimer’s disease. Detailed studies on the chemistry and spectroscopy of different curcumin-metal complexes would be helpful for future development of curcumin-metal complexes as imaging agents.

## 7. New Curcumin Delivery Systems

The water insolubility and low bioavailability of curcumin in cells have prompted researchers to develop new formulations based on biocompatible organic substances like liposomes, polyethylene glycols, biopolymers, cellulose, corn oil, hydrogels *etc.* [96,97,98]. Supramolecular assemblies of curcumin with cyclodextrins, and cucurbyturyl have also been reported. All these systems have not only shown improved water solubility but also increased curcumin bioavailability. In these systems, curcumin is solubilised by getting entrapped in hydrophobic pockets, mainly through hydrophobic interactions. Interestingly the fluorescence of curcumin gets is enhanced once solubilised in any of these systems, making it easy to estimate its binding efficiency. Due to their biocompatibility all these systems could be successfully investigated for anti-cancer activity in cancer cells, and *in vivo* systems, where significant increase in the anticancer activity due to improved bioavailability of curcumin was reported. Liposomal curcumin was found to be the best for improving the bioavailability of curcumin in cells [96] and products based on liposomal formulations are being marketed for different dietary applications of curcumin. Till recently the word nanocurucmin referred to curcumin-loaded organic formulations only. Since there are already several reviews published on organic nanoformulations, in this review, application of inorganic nano formulations in curcumin delivery is discussed.

With the same aim of improving anti-cancer activity of curcumin, in the last few years, researchers have been preparing formulations in which curcumin is bound to novel metal and oxide nanoparticles. Such systems can be easily manipulated for improved delivery, activity and specificity.

**Figure 2:**
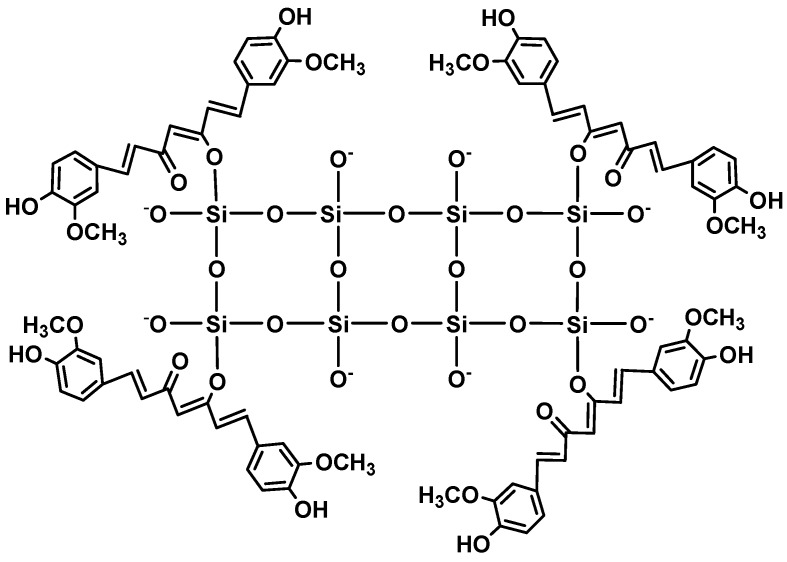
Curcumin-bound mesoporous silicon nanoparticles.

Mesoporous silica nanoparticles (MSN) are one of the most employed nanosystems for improving the bioavailability of poorly water soluble drugs [99,100,101,102,103,104,105,106,107]. Due to their ordered nanoporous structures, high surface areas, large pore volumes and high surface densities of hydroxyl groups, MSNs can be functionalized easily. They are biocompatible and they are commonly used in many biomedical applications. Curcumin binds covalently through a silicon-oxygen bond at the diketo moiety (Figure 2). Curcumin-loaded MSNs have been prepared and employed in several studies [99,100,101,102,103,104,105,106,107]. In these systems, curcumin release could be controlled for even up to several hours along with improvement in the stability and bioavailability of curcumin.

The fluorescence of curcumin is enhanced on MSN conjugation and therefore has the potential to be employed for imaging biomolecules/organelles. Novel cyclodextrin functionalized MSN have been found to be photothermally controlled on exposure to light to release curcumin on demand in zebrafish larve [102]. Large amount of curcumin could be loaded in to spherical microcapsules containing L-lysine, trisodiumcitrate and silica sol (colloidal suspension). These microcapsules could be triggered to release curcumin by adjusting the pH to acidic conditions [103,104]. MSN-curcumin conjugates, increased the cytotoxicity of curcumin in Hela cell lines and also in normal fibroblast cell lines [105]. They also increased photocytotoxicity of curcumin in human oral cancer cells, on exposure to light [106]. Other formulations like guanidine functionalized, and PEGylated MSN-curcumin conjugates showed improved bioavailability and controlled release of curcumin *in vitro* systems [107].

**Figure 3:**
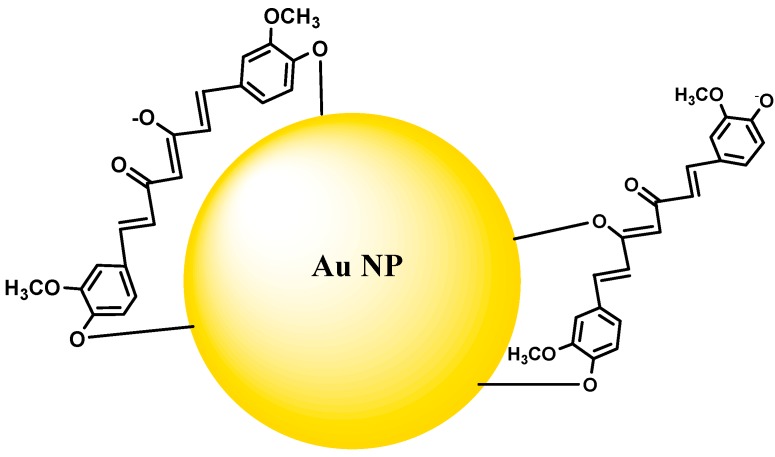
Gold nanoparticles capping by curcumin molecules.

Gold nanoparticle-based curcumin formulations have been prepared and reported recently. Gold nanoparticles find application in biology and medicine, for drug delivery, diagnosis and cancer treatment [108,109,110,111]. In a simple method, curcumin-gold composites were prepared by mixing alkaline curcumin solutions with gold salts, where the ionized curcumin acts both as a reducing agent and also as the capping agent [108]. In this case, both the phenolic-OH and enolic-OH donate hydrogen for reduction of Au^3+^ ions (Figure 3). Such gold-curcumin conjugates were reported to be hemocompatible and non-toxic. Singh *et al.* [109] also prepared gold-curcumin conjugates by mixng the gold salt with curcumin at high temperature. Such conjugates have been reported to exhibit antixodiant activity by DPPH assay. In another study, curcumin was first conjugated to hyaluronic acid (HA) and this conjugate is treated with gold salt, where HA acted as the reductant [110]. These gold-HA-curucmin composites were biocompatible and exhibited more cytotoxicity in cancer cell lines than pure curcumin. Other gold-curcumin composites were also prepared by conventional methods employing sodium citrate as reducing agent and polymeric systems as capping agents. Such systems can also be employed for the delivery of curcumin [111].

Curucmin-nanoconjugates of cobalt and silver nanoparticles have been shown to exhibit antimicrobial activity [112,113]. New antimicrobial films are being fabricated with silver nanocomposites along with curcumin for potentially treating microbial infections.

**Figure 4:**
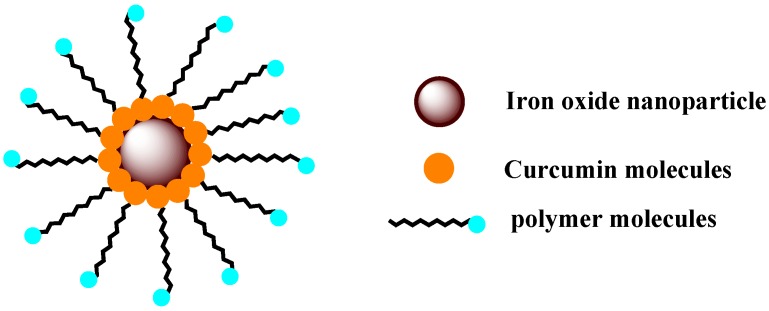
Polymer stabilized curcumin functionalized iron oxide magnetic nanoparticles.

Recently magnetic nanoparticles (MNP) are attracting the attention of researchers. MNP, mostly of iron oxide, are used as drug delivery systems and MRI contrasting agents [114,115,116,117]. They can be targeted magnetically and applied for local hyperthermia. Recently a few reports on preparation and charactirisation of MNP-curucmin conjugates are reported [114,115,116,117]. They have also been evaluated for diagnosis and anticancer activity. MNP is coated with pluronic polymers or other biopolymers to load hydrophobic drugs like curcumin (Figure 4). MNP-curucmin can be magnetically and selectively accumulated in cancer cells. In one such study, Yellapu *et al.* [115,116,117] prepared curcumin-loaded MNPs modified with cyclodextrin and reported inhibition effects in ovarian, breast, and prostate cancer cells and also in human pancreatic xenografts. MNP-curucmin has been found to cause apoptosis in MDA-MB-231 breast cancer cell lines along with loss of mitochondrial membrane potential and increased ROS production. MNP-curucmin improved the serum bioavailability by 2.5-fold. The formulation showed good MRI imaging characteristics. MNP formulations along with porous silica nanoparticles have also been employed to encapsulate curcumin, for efficient delivery of curcumin. Due to their selective accumulations and ability to cause hyperthermia, MNP-curcumin with suitable modifications are attracting the attention of many researchers for specific application in cancer therapy and diagnosis.

## 8. Conclusions and Future Directions

Curcumin is a specially gifted molecule provided by Mother-Nature to protect humans from chronic health problems. Looking at the simple chemical structure of curcumin, it is natural to presume that chemistry of curcumin is also very simple, however with increasing scientific understanding it appears to be more complex, unique and difficult to comprehend. It is a symmetric molecule abundant in turmeric with relatively high stability in natural form. It has an intense yellow color, that changes to deep red in basic pH solution. In simple aqueous and aqueous-organic solutions, it is susceptible to fast degradation, which increases as the basicity of the solutions increases, and also on exposure to sunlight. The metabolic products of curcumin are different from the degradation products, where O-conjugation and reduction are the important processes initiated through the enzymatic reactions. Interestingly unlike the degradation products, the metabolic products are much more difficult to synthesize in the laboratory. The presence of α,β-unsaturated structure makes curcumin participate in nucleophilic addition reactions with protein thiols and selenols, that play important role in modulating cellular oxidative stress. It is still not clear if these processes are reversible under physiological conditions. Future chemical research on these aspects is necessary to elucidate the kinetics and mechanism of all these reactions, so that a meaningful conclusion can be made on the role of these different processes in curcumin biology.

Recently there is a surge of activity on preparation and characterization of curcumin-metal complexes due to the strong affinity of β-diketo moiety as an efficient metal chelator. Although it is confirmed that curcumin reduces metal toxicity in living systems through complexation, the actual role of these metal complexes in curcumin biology appears to be complex and unclear. Detailed research is warranted on structure-activity evaluation of the curcumin-metal-complexes in solution.

Problems associated with curcumin bioavailability could be overcome to a great extent through formulations with natural biopolymers, which find practical application as nutritional products. Recent research is now focused on developing conjugates of curcumin with metal and metal oxide nanoparticles and some of these formulations have promising potential in nanomedicine with additional effects of inducing targeted hyperthermia in cancer cells. They are also attracting interest as diagnostic tools for Alzheimer’s disease and also as MRI contrast agents. Overall it appears that even though there has been significant progress in the chemistry of curcumin, a great deal can still be expected from chemists to exploit this divine natural product as a therapeutic remedy for many chronic diseases.
